# Supplementary material for: Adding context to the pneumococcal core genes using bioinformatic analysis of the intergenic pangenome of Streptococcus pneumoniae
Source: Front Bioinform. 2023 Feb 8;3:1074212. doi: 10.3389/fbinf.2023.1074212 (PMC9944727; doi:10.3389/fbinf.2023.1074212)
Supplement: Supplementary file 2 [file Table2.DOCX]

>csIGR1

AATTTTCTTTAAAAGAGTTTCTTTTTATACTTTTCTGAAGTGGTGACGGACGTCAGCAAAGTCCTTCGGACTTTCATGAC

TAAAATTTGAGCCTAAGGTCTCAAATTTTCCGCAGTTGGTACAATCACTTGTACCAACTTACACCACAGCGAAAAGTAT

TCTCTATGGGGCTCGCCTTGCTCGCCCAAATTCAGACAACCCTTTCCTTTCTGTGC

>csIGR2

AATTTTCTTCCTTGTTTTTTGATAGTTATTTTAGGTGGTGAGGGACGGTCGGAACTAATTTTCCAAAGATTTCATCTTTG

AAAAATGGATTTCCTGACCTCACAATTGAGCCTTGGTCTCAATTGTGCCCCCTTGCCAATCTACAGATTGGCAATCACA

CCACGGCAATAACTATCGCTATGTGAGCTCACTTTGTTCGCTCAGCGATTTTGTT

RNAstructure Fold MaxExpect Results for csIGR1

(Predict the lowest free energy structure and a set of low free energy structures for a sequence.)


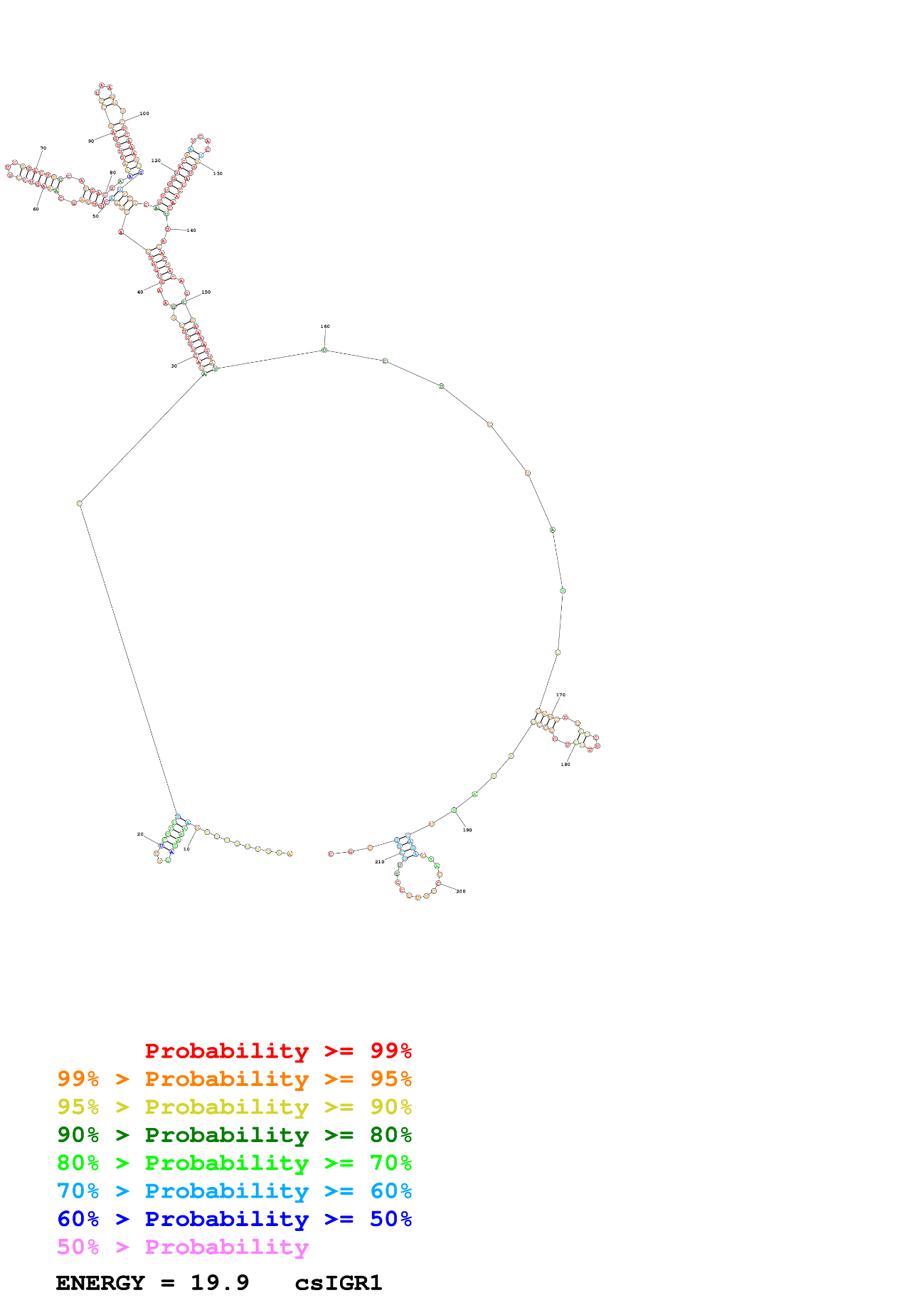


csIGR1 predicted secondary structure

RNAstructure ProbKnot Results for csIGR1

(Predict a secondary structure of probable base pairs, which might include pseudoknots.)


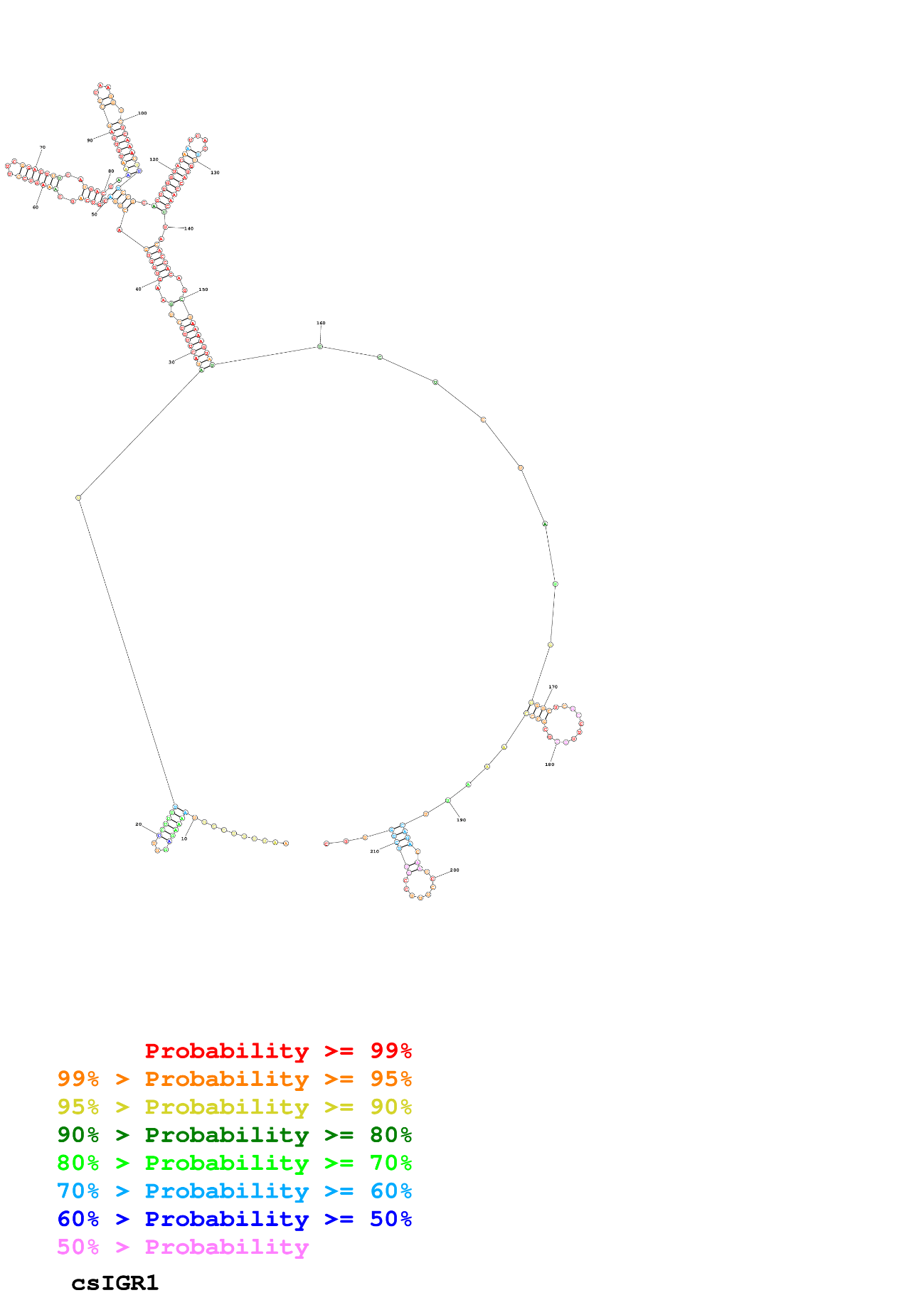


csIGR1 predicted secondary structure with pseudoknots included

RNAstructure Fold MaxExpect Results for csIGR2

(Predict the lowest free energy structure and a set of low free energy structures for a sequence.)


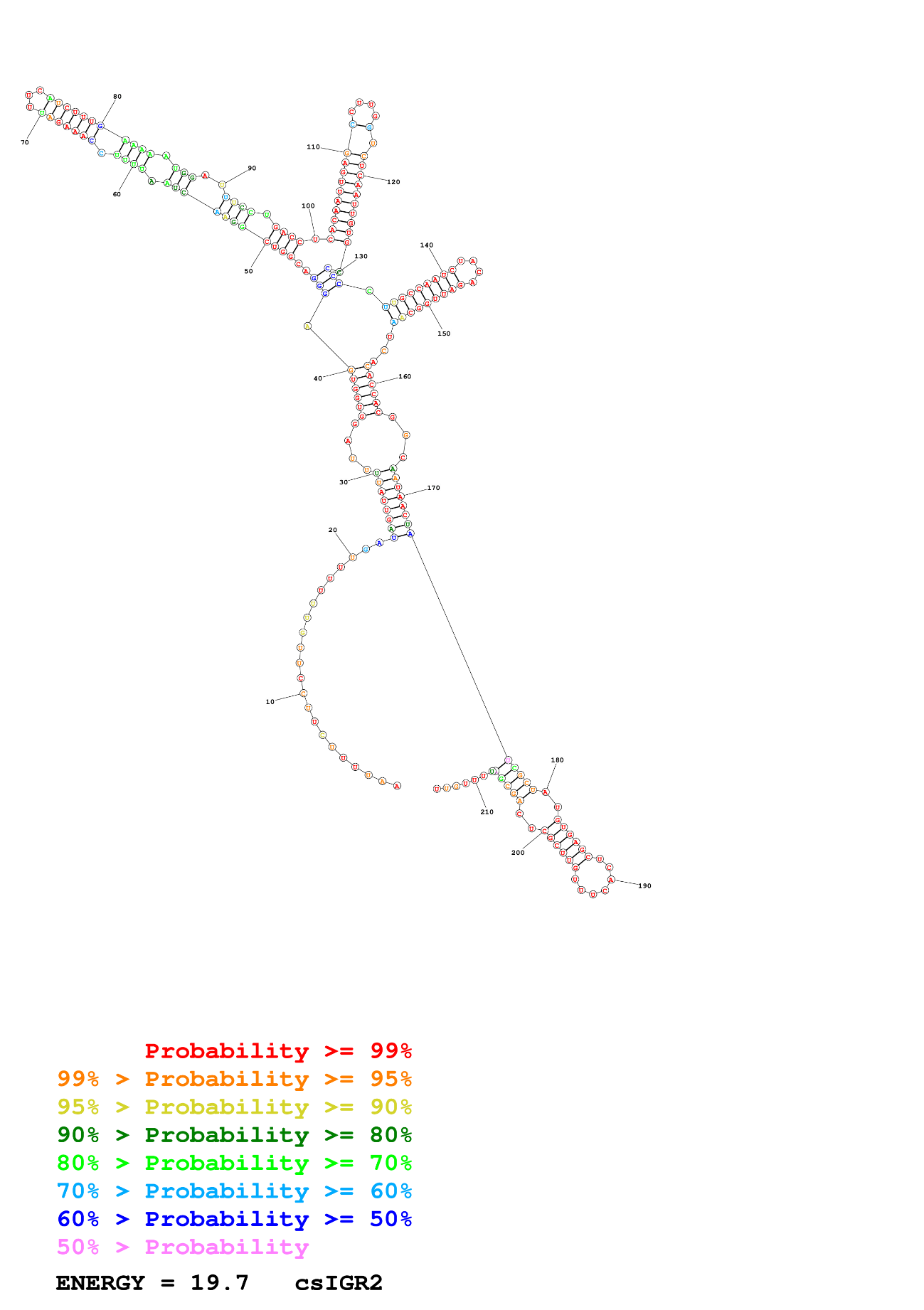


csIGR2 predicted secondary structure with pseudoknots included

RNAstructure ProbKnot Results for csIGR2

(Predict a secondary structure of probable base pairs, which might include pseudoknots.)


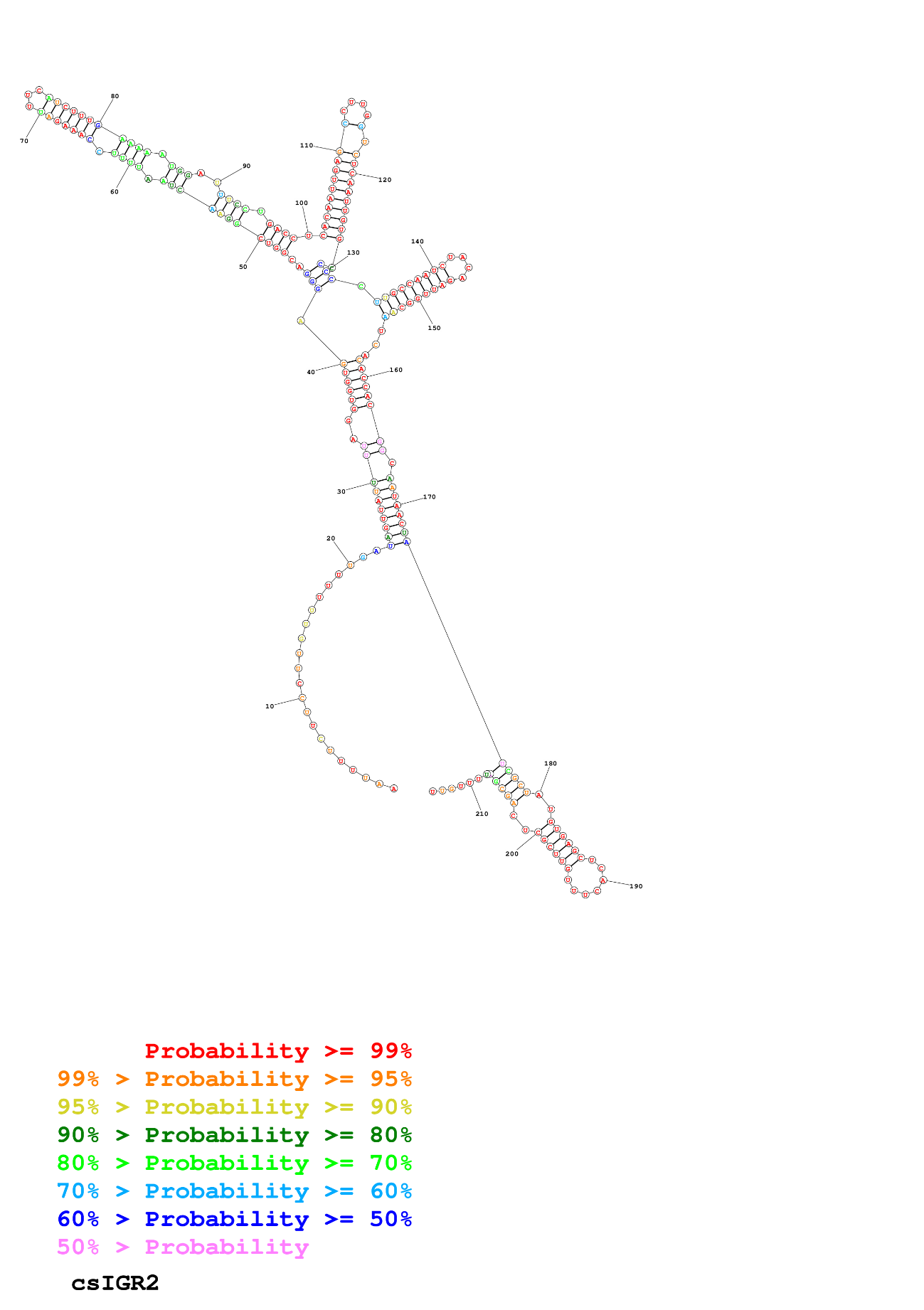


csIGR2 predicted secondary structure with pseudoknots included
