# Supplementary material for: Adding context to the pneumococcal core genes using bioinformatic analysis of the intergenic pangenome of Streptococcus pneumoniae
Source: Front Bioinform. 2023 Feb 8;3:1074212. doi: 10.3389/fbinf.2023.1074212 (PMC9944727; doi:10.3389/fbinf.2023.1074212)
Supplement: Supplementary file 4 [file Table4.DOCX]

library(tidyr)

library(dplyr)

##### Single isolate

#GCA_000014365.2 (D39), GCA_000007045.1 (R6), GCA_000006885.1 (Tigr4)

genome <- "GCA_000007045.1"

IGR <- read.csv("Path to IGR_presence_absence.csv", header=TRUE)

GENE <- read.csv("Path to gene_presence_absence.csv", header=TRUE)

IGR <- select(IGR, Gene, No..isolates, No..sequences, genome)

GENE <- select(GENE, Gene, No..isolates, No..sequences, genome)

#Removes any genes and IGRs not present in current genome

IGR <- IGR[!apply(IGR, 1, function(x) any(x=="")),]

GENE <- GENE[!apply(GENE, 1, function(x) any(x=="")),]

#Seperates the duplicated genes/IGRs into multiple rows

IGR <- separate_rows(IGR, genome, sep = "\t", convert = FALSE)

GENE <- separate_rows(GENE, genome, sep = "\t", convert = FALSE)

#Identifies tham as core or accessory

IGR$pan <- ifelse(IGR$No..isolates >= (84*0.95), "Core", "Accessory")

GENE$pan <- ifelse(GENE$No..isolates >= (84*0.95), "Core", "Accessory")

IGR <- table(IGR$pan)

IGR <- as.data.frame(IGR)

GENE <- table(GENE$pan)

GENE <- as.data.frame(GENE)

IGR$percent <- (100/sum(IGR$Freq))*IGR$Freq

IGR$percent <- round(IGR$percent, digits = 2)

GENE$percent <- (100/sum(GENE$Freq))*GENE$Freq

GENE$percent <- round(GENE$percent, digits = 2)

##### Percentage for entire species

library(data.table)

list <- read.csv("Path to IGR_presence_absence.csv", header=FALSE)

list <- transpose(list[1,])

list <- list$V1[grepl("^GCA", list$V1)]

IGR <- read.csv("Path to IGR_presence_absence.csv", header=TRUE)

GENE <- read.csv("Path to gene_presence_absence.csv", header=TRUE)

output <- data.frame()

for (i in list) {

genome <- i

IGR <- read.csv(“Path to IGR_presence_absence.csv", header=TRUE)

GENE <- read.csv("Path to gene_presence_absence.csv", header=TRUE)

IGR <- select(IGR, Gene, No..isolates, No..sequences, genome)

GENE <- select(GENE, Gene, No..isolates, No..sequences, genome)

#Removes any genes and IGRs not present in current genome

IGR <- IGR[!apply(IGR, 1, function(x) any(x=="")),]

GENE <- GENE[!apply(GENE, 1, function(x) any(x=="")),]

#Seperates the duplicated genes/IGRs into multiple rows

IGR <- separate_rows(IGR, genome, sep = "\t", convert = FALSE)

GENE <- separate_rows(GENE, genome, sep = "\t", convert = FALSE)

#Identifies tham as core or accessory

IGR$pan <- ifelse(IGR$No..isolates >= (84*0.95), "Core", "Accessory")

GENE$pan <- ifelse(GENE$No..isolates >= (84*0.95), "Core", "Accessory")

IGR <- table(IGR$pan)

IGR <- as.data.frame(IGR)

GENE <- table(GENE$pan)

GENE <- as.data.frame(GENE)

IGR$percent <- (100/sum(IGR$Freq))*IGR$Freq

IGR$percent <- round(IGR$percent, digits = 2)

GENE$percent <- (100/sum(GENE$Freq))*GENE$Freq

GENE$percent <- round(GENE$percent, digits = 2)

GENE <- transpose(as.data.frame(unlist(GENE)))

IGR <- transpose(as.data.frame(unlist(IGR)))

result <- merge(GENE, IGR, by = NULL)

output <- rbind(output, result)

}

output <- select(output, 3, 4, 5, 6, 9, 10, 11, 12)

colnames(output) <- c("Accessory_genes", "Core_genes","Accessory_genes_percent", "Core_genes_percent", "Accessory_IGRs", "Core_IGRs","Accessory_IGRs_percent", "Core_IGRs_percent")

outputMean <- as.data.frame(colMeans(output))

##### S. aureus data

library(data.table)

list <- read.csv("Path to IGR_presence_absence.csv", header=FALSE)

list <- transpose(list[1,])

list <- list$V1[grepl("^GCF", list$V1)]

IGR <- read.csv("Path to IGR_presence_absence.csv", header=TRUE)

GENE <- read.csv("Path to gene_presence_absence.csv", header=TRUE)

output <- data.frame()

for (i in list) {

genome <- i

IGR <- read.csv("Path to IGR_presence_absence.csv", header=TRUE)

GENE <- read.csv("Path to gene_presence_absence.csv", header=TRUE)

IGR <- select(IGR, Gene, No..isolates, No..sequences, genome)

GENE <- select(GENE, Gene, No..isolates, No..sequences, genome)

#Removes any genes and IGRs not present in current genome

IGR <- IGR[!apply(IGR, 1, function(x) any(x=="")),]

GENE <- GENE[!apply(GENE, 1, function(x) any(x=="")),]

#Seperates the duplicated genes/IGRs into multiple rows

IGR <- separate_rows(IGR, genome, sep = "\t", convert = FALSE)

GENE <- separate_rows(GENE, genome, sep = "\t", convert = FALSE)

#Identifies tham as core or accessory

IGR$pan <- ifelse(IGR$No..isolates >= (84*0.95), "Core", "Accessory")

GENE$pan <- ifelse(GENE$No..isolates >= (84*0.95), "Core", "Accessory")

IGR <- table(IGR$pan)

IGR <- as.data.frame(IGR)

GENE <- table(GENE$pan)

GENE <- as.data.frame(GENE)

IGR$percent <- (100/sum(IGR$Freq))*IGR$Freq

IGR$percent <- round(IGR$percent, digits = 2)

GENE$percent <- (100/sum(GENE$Freq))*GENE$Freq

GENE$percent <- round(GENE$percent, digits = 2)

GENE <- transpose(as.data.frame(unlist(GENE)))

IGR <- transpose(as.data.frame(unlist(IGR)))

result <- merge(GENE, IGR, by = NULL)

output <- rbind(output, result)

}

output <- select(output, 3, 4, 5, 6, 9, 10, 11, 12)

colnames(output) <- c("Accessory_genes", "Core_genes","Accessory_genes_percent", "Core_genes_percent", "Accessory_IGRs", "Core_IGRs","Accessory_IGRs_percent", "Core_IGRs_percent")

outputMean <- as.data.frame(colMeans(output))

##### Count average sequence or isolate

# pneumococcus

IGR <- read.csv("Path to IGR_presence_absence.csv", header=TRUE)

IGR <- IGR %>%

filter(No..isolates >= (84*0.95))

IGR <- select(IGR, 4,5,6)

outputIGR <- as.data.frame(colMeans(IGR))

GENE <- read.csv("Path to gene_presence_absence.csv", header=TRUE)

GENE <- GENE %>%

filter(No..isolates >= (84*0.95))

GENE <- select(GENE, 4,5,6)

outputGENE <- as.data.frame(colMeans(GENE))

# S. aureus

IGR <- read.csv("Path to IGR_presence_absence.csv", header=TRUE)

IGR <- IGR %>%

filter(No..isolates >= (84*0.95))

IGR <- select(IGR, 4,5,6)

outputIGR <- as.data.frame(colMeans(IGR))

GENE <- read.csv("Path to gene_presence_absence.csv", header=TRUE)

GENE <- GENE %>%

filter(No..isolates >= (84*0.95))

GENE <- select(GENE, 4,5,6)

outputGENE <- as.data.frame(colMeans(GENE))

#### Orientation plot

library(dplyr)

library(tidyr)

IGR <- read.csv("Path to IGR_presence_absence.csv", header=TRUE, na.strings=c("","NA"))

temp <- IGR[,15:98]

temp$Orientation <- do.call(coalesce, temp)

IGR <- full_join(IGR, temp)

IGR <- separate_rows(IGR, Orientation, sep = "\\t", convert = FALSE)

IGR <- separate(data = IGR, col = Orientation, into = c("Genome", "Gene_1", "Gene2", "Orientation"), sep = "_..._")

IGR <- select(IGR, Gene, No..isolates, Orientation)

rm(temp)

# Change the orientation names

IGR2 <- IGR

IGR2$Orientation <- gsub("CO_R|CO_F", "SR", IGR2$Orientation)

IGR2$Orientation <- gsub("DT", "NR", IGR2$Orientation)

IGR2$Orientation <- gsub("DP", "DR", IGR2$Orientation)

IGR2$pan <- ifelse(IGR2$No..isolates >= (84*0.95), "Core", "Accessory")

# Create plots

library(ggplot2)

library(ggdist)

ggplot(IGR2, aes(x = Orientation, y = No..isolates)) +

ggdist::stat_halfeye(adjust = .5, width = .6) +

coord_flip()

library(PupillometryR)

#group.colors <- c(SR = "#f5a489", NR = "#f5a489", DR ="#f5a489")

group.colors <- c(SR = "#76c2c3", NR = "#76c2c3", DR ="#76c2c3")

IGR2$Orientation <- factor(IGR2$Orientation , levels=c("SR", "DR", "NR"))

#scale = count (relative to size), scale = area (relative to proportion)

ggplot(IGR2, aes(x = Orientation, y = No..isolates, fill = Orientation)) +

geom_flat_violin(position = position_nudge(x = .2, y = 0), alpha = .8, scale = "area", color = NA) +

geom_point(aes(y = No..isolates, color = Orientation), position = position_jitter(width = .15), size = .5, alpha = 0.8) +

#geom_boxplot(width = .1, guides = FALSE, outlier.shape = NA, alpha = 0.5) +

coord_flip() +

guides(fill = "none") +

guides(color = "none") +

ylab("Isolates")+

xlab("")+

scale_fill_manual(values=group.colors) +

scale_color_manual(values=group.colors) +

theme(axis.line = element_line(colour = "grey"),

panel.grid.major = element_line(colour="white", size = (1)),

panel.grid.minor = element_line(size = (1), colour="white"),

panel.border = element_rect(colour = "grey", fill=NA, size=1),

panel.background = element_rect(fill = "#ecf0f1",

colour = "#ecf0f1",

size = 0.5, linetype = "solid"))

out_file=paste(out_dir, "/IGR_orientation_blue.png", sep="")

ggsave(out_file,

plot = last_plot(),

width = 6,

height = 5,

dpi = 600,

)

#### Table 1

library(dplyr)

library(data.table)

library(tidyr)

IGR <- read.csv("Path to IGR_presence_absence.csv", header=TRUE)

GENE <- read.csv("Path to gene_presence_absence.csv", header=TRUE)

genome <- "GCA_000006885.1"

core = 84*0.95

IGR <- select(IGR, Gene, No..isolates, genome)

IGR <- separate_rows(IGR, genome, sep = "\\t", convert = FALSE)

IGR <- separate(data = IGR, col = genome, into = c("Genome", "Gene1", "Gene2", "Orientation"), sep = "_._._")

IGR <- IGR[complete.cases(IGR), ]

#Remove DT

IGR <- IGR[!grepl("DT", IGR$Orientation),]

#Double DP an reverse every second instance

IGRtemp <- IGR[IGR$Orientation == "DP",]

IGRtemp <- IGRtemp[,c("Gene", "No..isolates", "Genome", "Gene2", "Gene1", "Orientation")]

colnames(IGRtemp) <- c("Gene", "No..isolates", "Genome", "Gene1", "Gene2", "Orientation")

IGR <- rbind(IGR, IGRtemp)

GENE <- select(GENE, Gene, No..isolates, genome)

GENE <- GENE[!apply(GENE, 1, function(x) any(x=="")),]

GENE <- separate_rows(GENE, genome, sep = "\t", convert = FALSE)

colnames(GENE)[3] <- "Gene2"

JOIN <- full_join(GENE, IGR, by = "Gene2", copy = FALSE, suffix = c(".gene", ".igr"),)

JOIN <- JOIN[complete.cases(JOIN), ]

JOIN <- JOIN %>%

mutate(

pan = case_when(

No..isolates.gene > core & No..isolates.igr > core ~ "CoreGene_CoreIGR",

No..isolates.gene < core & No..isolates.igr > core ~ "AccessoryGene_CoreIGR",

No..isolates.gene > core & No..isolates.igr < core ~ "CoreGene_AccessoryIGR",

No..isolates.gene < core & No..isolates.igr < core ~ "AccessoryGene_AccessoryIGR",

)

)

#Remove double occurence of genes in fvor of Core:core

JOIN <- JOIN[order(JOIN$pan, decreasing = TRUE),]

JOIN <- JOIN %>% distinct(Gene.gene, .keep_all = TRUE)

Output <- table(JOIN$pan)

Output <- as.data.frame(Output)

Output$percent <- 0

Output[1,3] = 100/(Output[1,2] + Output[2,2])*Output[1,2]

Output[2,3] = 100/(Output[1,2] + Output[2,2])*Output[2,2]

Output[3,3] = 100/(Output[3,2] + Output[4,2])*Output[3,2]

Output[4,3] = 100/(Output[3,2] + Output[4,2])*Output[4,2]

Output$percent <- round(Output$percent, digits = 2)

#### IGR_Gene linkage for entire pangenome

library(dplyr)

library(data.table)

library(tidyr)

list <- read.csv("Path to IGR_presence_absence.csv", header=FALSE)

list <- transpose(list[1,])

list <- list$V1[grepl("^GCA", list$V1)]

core = 84*0.95

Output <- data.frame()

result <- data.frame()

for (i in list) {

genome <- i

IGR <- read.csv("Path to IGR_presence_absence.csv", header=TRUE)

GENE <- read.csv("Path to gene_presence_absence.csv", header=TRUE)

IGR <- select(IGR, Gene, No..isolates, genome)

IGR <- separate_rows(IGR, genome, sep = "\\t", convert = FALSE)

IGR <- separate(data = IGR, col = genome, into = c("Genome", "Gene1", "Gene2", "Orientation"), sep = "_._._")

IGR <- IGR[complete.cases(IGR), ]

#Remove DT

IGR <- IGR[!grepl("DT", IGR$Orientation),]

#Double DP an reverse every second instance

IGRtemp <- IGR[IGR$Orientation == "DP",]

IGRtemp <- IGRtemp[,c("Gene", "No..isolates", "Genome", "Gene2", "Gene1", "Orientation")]

colnames(IGRtemp) <- c("Gene", "No..isolates", "Genome", "Gene1", "Gene2", "Orientation")

IGR <- rbind(IGR, IGRtemp)

GENE <- select(GENE, Gene, No..isolates, genome)

GENE <- GENE[!apply(GENE, 1, function(x) any(x=="")),]

GENE <- separate_rows(GENE, genome, sep = "\t", convert = FALSE)

colnames(GENE)[3] <- "Gene2"

JOIN <- full_join(GENE, IGR, by = "Gene2", copy = FALSE, suffix = c(".gene", ".igr"),)

JOIN <- JOIN[complete.cases(JOIN), ]

JOIN <- JOIN %>%

mutate(

pan = case_when(

No..isolates.gene > core & No..isolates.igr > core ~ "CoreGene_CoreIGR",

No..isolates.gene < core & No..isolates.igr > core ~ "AccessoryGene_CoreIGR",

No..isolates.gene > core & No..isolates.igr < core ~ "CoreGene_AccessoryIGR",

No..isolates.gene < core & No..isolates.igr < core ~ "AccessoryGene_AccessoryIGR",

)

)

#Remove double occurence of genes in fvor of Core:core

JOIN <- JOIN[order(JOIN$pan, decreasing = TRUE),]

JOIN <- JOIN %>% distinct(Gene.gene, .keep_all = TRUE)

Output <- table(JOIN$pan)

Output <- as.data.frame(Output)

Output$percent <- 0

Output[1,3] = 100/(Output[1,2] + Output[2,2])*Output[1,2]

Output[2,3] = 100/(Output[1,2] + Output[2,2])*Output[2,2]

Output[3,3] = 100/(Output[3,2] + Output[4,2])*Output[3,2]

Output[4,3] = 100/(Output[3,2] + Output[4,2])*Output[4,2]

Output <- round(Output$percent, digits = 2)

result <- rbind(result, Output)

}

colnames(result) <- c("Accessory gene:accessory IGR", "Accessory gene:core IGR", "Core gene:accessory IGR", "Core gene:core IGR")

resultMean <- as.data.frame(round(colMeans(result), digits =2))

#### Table 2 - Percentage for entire species

library(tidyr)

library(dplyr)

library(data.table)

list <- read.csv("G:/Bioinformatics/Paper_2/Roary/piggy_out_roary_comparsion/IGR_presence_absence.csv", header=FALSE)

list <- transpose(list[1,])

list <- list$V1[grepl("^GCA", list$V1)]

IGR <- read.csv("Path to piggy_out_roary_comparsion/IGR_presence_absence.csv", header=TRUE)

GENE <- read.csv("Path to gene_presence_absence.csv", header=TRUE)

output <- data.frame()

for (i in list) {

genome <- i

IGR <- read.csv("Path to IGR_presence_absence.csv", header=TRUE)

GENE <- read.csv("Path to gene_presence_absence.csv", header=TRUE)

IGR <- select(IGR, Gene, No..isolates, No..sequences, genome)

GENE <- select(GENE, Gene, No..isolates, No..sequences, genome)

#Removes any genes and IGRs not present in current genome

IGR <- IGR[!apply(IGR, 1, function(x) any(x=="")),]

GENE <- GENE[!apply(GENE, 1, function(x) any(x=="")),]

#Seperates the duplicated genes/IGRs into multiple rows

IGR <- separate_rows(IGR, genome, sep = "\t", convert = FALSE)

GENE <- separate_rows(GENE, genome, sep = "\t", convert = FALSE)

#Identifies tham as core or accessory

IGR$pan <- ifelse(IGR$No..isolates >= (84*0.95), "Core", "Accessory")

GENE$pan <- ifelse(GENE$No..isolates >= (84*0.95), "Core", "Accessory")

IGR <- table(IGR$pan)

IGR <- as.data.frame(IGR)

GENE <- table(GENE$pan)

GENE <- as.data.frame(GENE)

IGR$percent <- (100/sum(IGR$Freq))*IGR$Freq

IGR$percent <- round(IGR$percent, digits = 2)

GENE$percent <- (100/sum(GENE$Freq))*GENE$Freq

GENE$percent <- round(GENE$percent, digits = 2)

GENE <- transpose(as.data.frame(unlist(GENE)))

IGR <- transpose(as.data.frame(unlist(IGR)))

result <- merge(GENE, IGR, by = NULL)

print(result)

output <- rbind(output, result)

}

output <- select(output, 3, 4, 5, 6, 9, 10, 11, 12)

colnames(output) <- c("Accessory_genes", "Core_genes","Accessory_genes_percent", "Core_genes_percent", "Accessory_IGRs", "Core_IGRs","Accessory_IGRs_percent", "Core_IGRs_percent")

outputMean <- as.data.frame(colMeans(output))

##### Count average sequence or isolate

IGR <- read.csv("Path to IGR_presence_absence.csv", header=TRUE)

IGR <- IGR %>%

filter(No..isolates >= (84*0.95))

IGR <- select(IGR, 4,5,6)

outputIGR <- as.data.frame(colMeans(IGR))

GENE <- read.csv("Path to gene_presence_absence.csv", header=TRUE)

GENE <- GENE %>%

filter(No..isolates >= (84*0.95))

GENE <- select(GENE, 4,5,6)

outputGENE <- as.data.frame(colMeans(GENE))
